# Changes in brain and behavior during food-based decision-making following treatment of anorexia nervosa

**DOI:** 10.1186/s40337-021-00402-y

**Published:** 2021-04-17

**Authors:** Karin Foerde, B. Timothy Walsh, Maya Dalack, Nathaniel Daw, Daphna Shohamy, Joanna E. Steinglass

**Affiliations:** 1 New York State Psychiatric Institute, 1051 Riverside Drive, Unit 98, New York, NY 10032 USA; 2 Psychiatry Department, Columbia University Irving Medical Center, 1051 Riverside Drive, Unit 98, New York, NY 10032 USA; 3 Princeton Neuroscience Institute, Department of Psychology, Princeton University, Princeton, NJ 08540 USA; 4 Psychology Department and Zuckerman Mind Brain and Behavior Institute, Columbia University, 3227 Broadway, New York, NY 10027 USA

**Keywords:** Anorexia nervosa, Treatment, fMRI, Neuroscience, Longitudinal, Eating behavior

## Abstract

**Background:**

Anorexia nervosa is a severe illness with a high mortality rate, driven in large part by severe and persistent restriction of food intake. A critical challenge is to identify brain mechanisms associated with maladaptive eating behavior and whether they change with treatment. This study tested whether food choice-related caudate activation in anorexia nervosa changes with treatment.

**Methods:**

Healthy women (*n* = 29) and women hospitalized with anorexia nervosa (*n* = 24), ages 18 to 40 years, completed a Food Choice Task during fMRI scanning at two timepoints. Among patients, procedures occurred upon hospital admission (Time 1) and again after patients had gained to normal weight (Time 2). Healthy controls were tested twice at an interval group-matched to patients. Choice-related caudate activation was assessed at each timepoint, using parametric analyses in an a priori region of interest.

**Results:**

Among patients, the proportion of high-fat foods selected did not change over time (*p’*s > 0.47), but decreased neural activity in the caudate after treatment was associated with increased selection of high-fat foods (r_23_ = − 0.43, *p* = 0.037). Choice-related caudate activation differed among women with anorexia nervosa vs healthy control women at Time 1 (healthy control: *M* = 0.15 ± 0.87, anorexia nervosa: *M* = 0.70 ± 1.1, t_51_ = − 2.05, *p* = 0.045), but not at Time 2 (healthy control: *M* = 0.18 ± 1.0, anorexia nervosa: *M* = 0.37 ± 0.99, t_51_ = − 0.694, *p* = 0.49).

**Conclusions:**

Caudate activity was more strongly associated with decisions about food among individuals with anorexia nervosa relative to healthy comparison individuals prior to treatment, and decreases in caudate engagement among individuals with anorexia nervosa undergoing treatment were associated with increases in high-fat food choices. The findings underscore the need for treatment development that more successfully alters both eating behavior and the neural mechanisms that guide it.

**Supplementary Information:**

The online version contains supplementary material available at 10.1186/s40337-021-00402-y.

## Plain English summary

Treatment that leads to full weight restoration in anorexia nervosa (AN) is accompanied by improvements in psychological symptoms. Yet, restrictive eating does not improve. Prior studies have shown that different neural circuits are engaged when deciding what to eat for patients with AN, compared with healthy controls. Here, we tested whether there were changes in brain activation associated with eating after weight restoration. Overall, food choices did not change with weight restoration, again showing that restrictive eating is resistant to change. However, changes among patients with AN showed a significant association between increased selection of high-fat foods and decreased activity in the caudate, with treatment. These findings highlight the importance of specific brain systems related to eating in the treatment of AN.

## Background

The first step in the treatment of anorexia nervosa (AN) is weight restoration [1]. Behaviorally-based structured programs incentivizing patients with AN to consume adequate calories and normative macronutrient composition (i.e., at least 30% kcal from dietary fat) have high success rates [2]. Yet, relapse and rehospitalization of adults with AN are common, even after full weight restoration [3]. A central behavioral disturbance that contributes to relapse and chronicity of illness is the persistent restriction of food intake [4]. The neural mechanisms that underlie decisions about eating in AN have become a focus of recent research, providing an opportunity to test empirically how such mechanisms are affected by treatment. Increased understanding of the effects of treatment on neural mechanisms underlying the decision of what to eat may be useful in identifying biomarkers and new treatment targets.

In standard treatment programs for AN, renourishment is accomplished by providing appropriate nutrition, supervision around meals, and behavioral and psychological therapy to promote healthy eating and weight restoration [2]. Success rates for inpatient treatment are high in terms of weight normalization, resolution of medical instability, and improvement in associated psychological symptoms including depression and anxiety [5]. In these structured settings, individuals with AN are able to consume the ~ 4000 kcal per day that are required to gain weight at an appropriate pace. And yet, as soon as treatment incentives are removed after hospital discharge, these same individuals struggle to consume adequate nutrition and often lose weight [6, 7]. In two laboratory meal studies, hospitalized patients with AN showed significant improvement in psychological measures after successful weight restoration, but still consumed significantly fewer calories and less fat than healthy volunteers at both timepoints [8, 9]. The persistence of this maladaptive eating pattern indicates that it is important to understand this behavior.

Decision science has recently yielded advances in understanding the behavioral, computational, and neural mechanisms of choices [10–13]. Much of this research has involved food choices, partly for convenience of experimental design, leading to insights of particular relevance for eating disorders. Choice is a complex phenomenon that incorporates multiple cognitive and neural processes, such as attention, valuation, and action selection [14]. In decisions about what to eat, healthiness and tastiness have repeatedly been shown to be distinguishable values that are integrated into choice selection [12, 15–17]. The neurobiology of food choice has been shown to involve regions associated with value representations ventromedial prefrontal cortex (VMPFC) and self-control dorsolateral prefrontal cortex (DLPFC) [12]. The neural mechanisms of decision-making, and decisions about what foods to eat, have received relatively little attention in AN. Yet understanding the neural bases of maladaptive eating behavior may be important in order to improve treatment.

In two studies from our group, food choices made by adult women with AN were associated with neural activity in the dorsal striatum (specifically, the anterior caudate) [18, 19], which differed from healthy peers. This research used a food choice task that has been demonstrated to capture the maladaptive restrictive choices of patients with AN [20, 21] with both the restricting and the binge-eating/purging subtypes [22]. Task-based choices have been related to actual food intake in a laboratory meal [19], and test-retest reliability of the task has been demonstrated among healthy individuals [23]. However, changes in the neural underpinnings of decision-making about food have not been examined after weight restoration treatment.

Here, we examined whether food choice-related activation in regions of interest identified in previous studies [12, 19, 24], primarily the anterior caudate, VMPFC, and DLPFC changed, with treatment. Patients with AN participated in a Food Choice Task during fMRI scanning twice, once at admission for inpatient weight restoration treatment, and again after treatment. Change with treatment was compared with a group of healthy comparison (HC) women, who were studied twice, at an interval matched to the AN group. We hypothesized that individuals with AN would show greater choice-related activation of the anterior caudate as compared with HC and that this difference between groups would diminish after patients received weight restoration treatment, whereas groups would not differ in choice-related activation in the VMPFC and DLPFC. We additionally explored differences between groups and changes with treatment in choice-related, as well as tastiness and healthiness rating-related, activation in whole-brain analyses.

## Methods

### Participants

Participants were female HC and female inpatients with AN at the New York State Psychiatric Institute (NYSPI). Eligible patients met DSM-5 [25] criteria for AN at the time of admission assessed via Eating Disorders Assessment for DSM-5 [26]. HC were included if they had no current or lifetime psychiatric illness (including an eating disorder) and had a body mass index (BMI) between 18.5 and 25.0 kg/m^2^. In both groups, individuals were excluded if they were taking psychotropic medication, had a known history of a neurological disorder or injury, had any contraindication to MRI, or were pregnant. All participants were right-handed, between the ages 18–40 years, and had an estimated IQ > 80. Participants were enrolled between November 2015 and August 2018.

Procedures occurred twice, once upon admission (Time 1) and once again after weight restoration (Time 2) for individuals with AN, and within 1–2 months for HC, to match the treatment time frame. Inpatient treatment at NYSPI is provided at no cost and consists of a behaviorally-based program aimed at full weight restoration [2] with a BMI goal between 19.5–20.5 kg/m^2^. HC were compensated $150 for participation at each timepoint ($300 for both).

Only data from individuals who completed the Choice phase of the Food Choice Task during neuroimaging at both timepoints were analyzed. Time 1 data from these participants were included in one previous publication of food choice, focused on individuals across a spectrum of restrictive eating [18]. Of 31 HC, two were excluded from analyses due to loss of neuroimaging data (1 scanner error, 1 excessive motion) leading to a study sample of 29 HC. Of 26 AN, two were excluded due to a loss of task responses during neuroimaging, leaving a study sample of 24 AN (9 restricting subtype and 15 binge-eating/purging subtype). Two HC were additionally excluded from the Healthiness rating phase (1 due to excessive motion, 1 due to image artifact), and one AN was additionally excluded from the Healthiness and Tastiness rating phase due to excessive motion.

This study was approved by the NYSPI Institutional Review Board and all participants provided written informed consent.

### Procedure

Height was measured on a Seca 240 wall-mounted stadiometer. Weight was measured in the morning at each timepoint on a Detecto balance-beam scale.

Participants were given a standardized lunch (4 oz. turkey breast, 2 slices whole wheat bread, 1 packet mayonnaise, Nutrigrain bar, and 8 oz. water, ~ 500 kcal) at noon. At Time 2, for patients with AN, the post-treatment standardized lunch was increased in calories, such that the percent of the daily caloric prescription was the same at both timepoints. At approximately 2 pm, participants were brought to the NYSPI MR Unit for the Food Choice Task with fMRI scanning. At the end of the task, the participant was served a snack based on one of her choices in the task (see below), observed by staff. Procedures at Time 2 were identical.

### Food choice task with fMRI (Fig. 1)

**Fig. 1:**
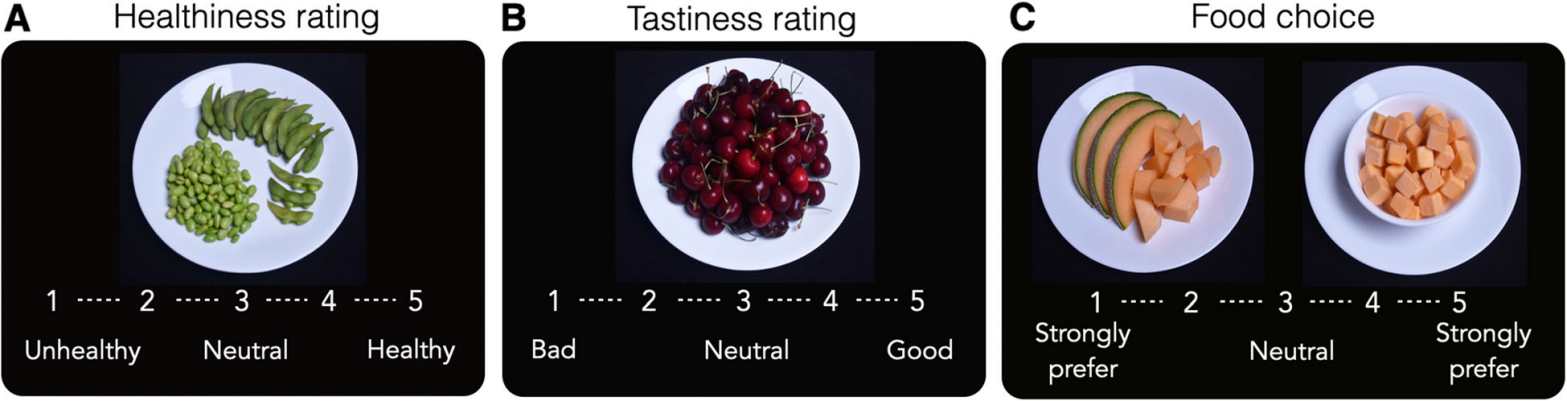
Food Choice Task. The task consisted of two rating phases and a choice phase. In each phase participants made decisions about 76 food items. On each trial, the food stimulus was presented for 4 s, during which the participant made her response. **a** Healthiness rating phase. Participants rated the healthiness of each food item on five-point Likert scale from Bad to Good (or Good to Bad, counterbalanced across participants). **b** Tastiness rating phase. Participants rated the tastiness of each food item on five-point Likert scale from Unhealthy to Healthy (or Healthy to Unhealthy, counterbalanced across participants). **c** Choice phase. On each trial, participants indicated their preference for a changing food item (shown on the right) relative to a repeated reference item (previously rated neutral on healthiness and tastiness; shown on the left). After task completion, one Choice trial was randomly selected and the participant served a snack-sized portion of the food selected on that trial

Food Choice Task procedures have been described previously [19]. The task consisted of three phases: Healthiness rating, Tastiness rating, and a Choice block (Fig. 1). In each block, participants rated 76 foods on a 5-point scale. Half of the foods were classified as “high-fat” (> 30% of the calories from fat; images and macronutrient information available at Columbia Academic Commons: 10.7916/d8-497c-2724 [17]. The rating scale appeared at the bottom of the screen for each item and participants were instructed that they could rate it as “neutral” or along the scale. For the Healthiness block, the anchors were “Unhealthy” to “Healthy.” For the Tastiness block, the anchors were “Bad” to “Good.” For the Tastiness block, participants were additionally instructed to rate it *only* for tastiness. All task parameters (including order of the rating scale) were counterbalanced and randomized across participants. After completion of the rating scales, a “Reference” item was selected for each participant that had been rated by her as “Neutral” on both Healthiness and Tastiness. If no item neutral on both scales was identified, an item was selected that was rated neutral on Healthiness and 1 point in the positive direction on Tastiness, in order to minimize biasing choices based on taste value. In the Choice block, participants made a selection by indicating whether they ‘Strongly Preferred’ or ‘Preferred’ the Reference item (which was constant) or the other food (which varied on each trial) using a 5-point scale. To ensure that responses reflected true preferences, participants were told that they would be served one of their choices at the conclusion of the task. One Choice trial was randomly selected, and the participant received a snack-sized portion of the item chosen on that trial.

In all blocks, the food stimulus was presented for 4 s on each trial, during which the participant made her response. Each trial was followed by a fixation cross inter-trial interval (ITI). The duration of ITIs was jittered for optimization of event-related fMRI design. Stimulus presentation sequence and timing were optimized using the optseq2 algorithm (http://surfer.nmr.mgh. harvard.edu/optseq/). Each learning run lasted 480 s. Mean ITI = 2.3 s, median = 2 s, and range = 1–10 s, across all three phases. All task phases were presented using Matlab and the Psychophysics toolbox [27].

Whole-brain imaging data were acquired on a GE 3 T MR750 scanner with a 32-channel phased-array head coil. Structural images were collected using a high-resolution T1-weighted BRAVO pulse sequence (1 × 1 × 1 mm voxel size) for image registration. Functional images were collected using a gradient echo T2*-weighted echoplanar (EPI) sequence with blood oxygenation level-dependent (BOLD) contrast (TR = 2000 ms, TE = 19 ms, flipangle = 77, 3 × 3 × 3 mm voxel size, 45 contiguous axial slices, FOV = 19.2, interleaved acquisition). Each Food Choice Task run consisted of 240 volumes.

### Data analysis

Demographic and clinical characteristics were compared using Student’s t-tests and chi-square, as appropriate. Repeated measures ANOVA were used to test for significance of change over time within each group. Tests were two-tailed unless otherwise specified. Analyses were conducted in JASP [28] (https://jasp-stats.org), and alpha was set at *p* = 0.05.

#### Food choice task

Rating and choice data were analyzed using multilevel regression models lme4 linear mixed effects package for R [29]. For the Choice phase, responses on the 5-point scale were converted to binary ‘Yes’ (1) or ‘No’ (0) preferences for the trial-unique food versus the Reference item, and neutral responses were omitted from analyses. Binomial choice data were modelled with multilevel logistic regression, in which participant choice (selection of the trial-unique food item over the reference food) was the dependent variable. Continuous outcome rating data from the Healthiness and Tastiness phases were modelled using multilevel linear regression. To assess relationships between ratings and choices, participant choice (selection of the trial-unique food item over the reference food) was the dependent variable and (z-scored) healthiness and tastiness ratings entered as independent variables. The relationship between Healthiness and Tastiness was assessed with Healthiness ratings as the dependent variable and (z-scored) Tastiness ratings as the independent variable. In all analyses, Group (HC/AN, coded as − 1/1), Food type (low-fat/high-fat, coded as − 1/1), and Time (Time 1/Time 2, coded as − 1/1) were entered as independent variables, and models included by-subject random intercepts and slopes and by-item (food images) random intercepts [30, 31].

Correlations between brain, behavior, and demographic measures were assessed using Pearson correlation. Robust regression was implemented as needed due to presence of outlier data points using the rlm function in the MASS package with bisquare weighting [32].

#### fMRI analyses

Imaging data were converted from DICOM to NIFTI format and preprocessed and analyzed using the FSL (http://fsl.fmrib.ox.ac.uk/fsl/) package version 6 (FMRIB’s Software Library; Oxford Centre for Functional Resonance Imaging of the Brain, FMRIB) [33].

##### Image pre-processing

Functional images were aligned using the MCFLIRT tool [34] and the six scan-to-scan head motion parameters estimated during motion correction obtained. The skull was removed from functional images using the brain extraction tool (BET) [35] and from structural images using Freesurfer [36, 37]. Spatial smoothing was applied with a Gaussian kernel of 5 mm (FWHM). Data and design matrix were high-pass filtered with a cutoff period of 100 s. After analysis at the individual level, the results were normalized to a standard template: Functional images were first aligned to the T1-weighted image using a boundary-based registration method implemented in FSL6 (BBR) and then the structural image to the standard MNI152 2-mm template using FLIRT (12 degrees of freedom) and FNIRT (10-mm warp resolution) [34, 38].

##### Analyses

At the level of individual participants, each event was convolved with a canonical hemodynamic response function (except added confound regressors, see below) and entered into a general linear model (GLM). The temporal derivative of each regressor (except added confound regressors) was also included in the model. To account for any residual effects of subject movement, we included the six scan-to-scan head motion parameters estimated during motion correction as well as framewise displacement (FD) and root mean square intensity difference from one volume to the next (DVARS) [39] as confound regressors. In addition, volumes with FD and DVARS exceeding a threshold of 0.5 were modeled out by adding a single time point regressor for each volume to be ‘dropped’ from analysis [40]. Runs for which more than 25% of volumes were dropped were excluded from analysis: one Tastiness rating run at Time 1 (AN), two Healthiness rating runs at Time 2 (1 HC, 1 AN). The number of dropped volumes did not differ between groups (ps > 0.05, Supplemental Table S1).

Parametric analysis of food choices and ratings were conducted (rating-related results in Supplemental Figure S1) [19]. Each person’s choices were normalized to their own response range; analyses were therefore not biased by overall differences in choice preferences. The GLMs for the choice and rating phases included the following regressors: onsets for each trial on which a response was made (i), onsets for each trial on which a response was made parametrically modulated by the (demeaned) rating on that trial (ii) and the (demeaned) response time on that trial (iii), and onsets for missed trials (iv). Regressors i-iii were modeled with duration equal to the response time on each trial, and regressor iv with duration equal to the trial length (4 s). Motion and confound regressors were included as outlined above. Linear contrasts were performed on specific comparisons of interest. These contrasts were used for mixed-effects group analyses using FSL’s FLAME 1 (FMRIB’s local analysis of mixed effects) tool, using two-sample unpaired *t-*tests to compare groups and paired *t*-tests to compare changes between Time 1 and Time 2.

#### ROI analyses

Based on a previous study indicating involvement of the right anterior caudate in food choices [19], an anatomical region of interest (ROI) was obtained from the Harvard-Oxford probabilistic atlas included in FSL, thresholded at 25% probability and anterior to y = 0 (Fig. 3a). Additional ROIs were included based on their implication in food choices in healthy individuals, with the VMPFC involved in value-based decisions and the DLPFC implicated in self-control [12]. Moreover, VMPFC regions (e.g., OFC) have been implicated in studies of decision making in AN [41] and DLPFC has been used as a target for neuromodulation (rTMS) intervention in AN [20]. Six-mm spheres were created centered on MNI coordinates taken from a previous study using the Food Choice task [12]: VMPFC (MNI = [3 51 3]; Fig. 3b) and DLPFC (MNI = [− 48 15 24]; Fig. 3c).

#### Whole-brain analyses

Whole-brain higher-level analyses were thresholded using clusters determined by *Z* > 3.1 and a whole-brain corrected, FWE cluster significance threshold of *p* = 0.05 [42]. For exploratory purposes, we additionally considered analyses thresholded at *Z* > 2.3, FWE cluster significance threshold of *p* = 0.05 [43].

Results are displayed on a study-specific mean anatomical image resulting from averaging all participants’ normalized high-resolution structural images.

## Results

Clinical characteristics of participants are presented in Table 1. Psychological measures differed between individuals with AN and HC at both timepoints, and the AN group showed improvement in all psychological measures and BMI from Time 1 to Time 2. At Time 2, AN and HC groups were well matched on BMI (t_51_ = 1.29, *p* = 0.20). The average time between scans was similar in both groups (mean_HC_: 49 ± 21 days; mean_AN_: 58 ± 21 days, t_51_ = − 1.4, *p* = 0.17).

**Table 1:** Demographics and clinical characteristics of participants

	Time 1						Time 2						Time 1 versus Time 2			
	HC (n = 29)		AN (n = 24)				HC		AN				HC		AN	
	M	SD	M	SD	t	*p*	M	SD	M	SD	t	*p*	t	*p*	t	*p*
Age (years)	25.8	5.2	26.9	6.5	−0.7	*0.50*										
Caucasian (n,%)	22	76%	16	67%	0.55^a^	*0.46*										
Estimated IQ	117.3	13.4	112.0	9.5	1.7	*0.11*										
LNS	12.0	3.1	11.7	2.4	0.4	*0.67*										
BMI (kg/m2)	21.0	1.4	16.3	1.9	10.3	*< .001*	21.0	1.6	20.5	0.9	1.4	*0.17*	−0.33	*0.75*	−13.4	*< .001*
Duration of Illness (years)			8.6	6.9												
EDE-Q Global	0.4	0.5	4.3	1.7	− 12.2	*< .001*	0.4	0.4	2.8	1.3	−9.1	*< .001*	−0.54	*0.6*	6.6	*< .001*
TFEQ-Restraint	5.9	4.2	17.4	3.6	−10.6	*< .001*	6.1	4.8	14.2	5.3	−5.9	*< .001*	−0.43	*0.67*	3.9	*< .001*
BDI	2.1	2.3	31.1	13.4	−11.5	*< .001*	3.1	2.5	17.4	14.4	−5.2	*< .001*	−1.94	*0.06*	5.3	*< .001*
STAI(T)	32.6	6.5	62.9	11.4	−12.2	*< .001*	32.9	8.2	56.0	12.2	−8.2	*< .001*	−0.26	*0.8*	3.6	*0.003*

^a^Chi square statistic

Missing data: BDI is missing from 1 individual with AN

*AN* anorexia Nervosa, *BDI* beck depression index [44], *BMI* body mass index, *EDE-Q* eating disorder examination-questionnaire version [45], *HC* healthy control, *LNS* letter number sequence from the Weschler Adult Intelligence Scale [46], *STAI(T)* Spielberger trait anxiety inventory [47], *TFEQ-Restraint* Three Factor Eating Questionnaire Restraint subscale [48] Estimated IQ was assessed with the Wechsler Abbreviated Scale of Intelligence [49]

### Food choice task behavior

All participants were able to respond within the response window, indicated by response rates exceeding 96% in all task phases and in both groups. Response rates did not differ between groups (ps > 0.25).

**Fig. 2:**
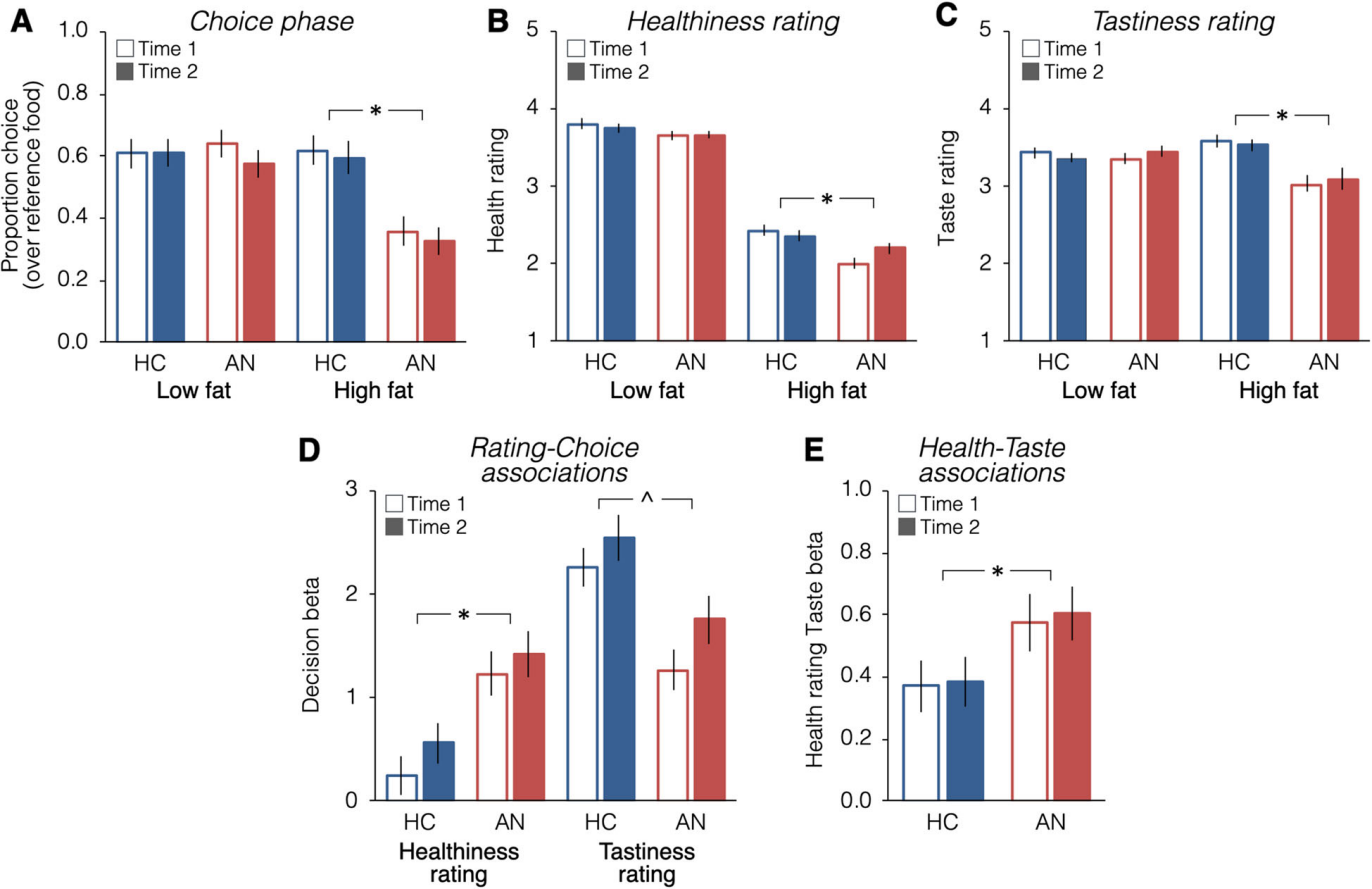
Food choice task behavior at Time 1 and Time 2. **a** High-fat and low-fat food choices did not change significantly from Time 1 to Time 2 (ps > 0.37). The AN group made fewer high-fat, but not low-fat, food choices before and after treatment (Supplemental Table S2). **b** Overall low-fat items were rated higher on healthiness than high-fat items. High-fat foods were rated as lower in healthiness than low-fat foods overall and the groups differed significantly (Supplemental Table S3). **c** The AN group rated high-fat foods specifically lower in tastiness than did the HC group and this did not change with treatment (Supplemental Table S4). **d** Logistic regression of Healthiness and Tastiness ratings on choice. Choice was influenced more by tastiness among HC than AN, whereas choice was influenced more by healthiness among AN relative to HC (Supplemental Table S5). **e** Regression of Healthiness on Tastiness ratings. Ratings were more strongly associated among AN than HC (Supplemental Table S6). * indicates *p* < 0.05. ^ indicates significant Group difference and significant difference between Time 1 and Time 2 in both groups

Choices of high-fat and low-fat foods (proportion of trials chosen over the Reference food) did not change significantly between Time 1 and Time 2 (ps > 0.16) (Fig. 2a). On average, the AN group chose high-fat foods less often than the HC group at both time points (Group X Food type interaction: Est = − 0.42, z = − 4.72, *p* < 0.00001; Supplemental Table S2; significant effect of Group for high-fat foods: Est = − 0.14, z = − 4.61, *p* < 0.0001, but not for low-fat foods: Est = − 0.0008, z = − 0.03, *p* = 0.97).

Healthiness ratings did not change significantly with treatment (ps > 0.05, Fig. 2b, Supplemental Table S3). High-fat foods were rated lower in healthiness than low-fat foods overall (Est: − 0.75, t_80.3_ = − 6.70, *p* = 2.6 × 10^− 9^) and significantly more so among individuals with AN (Group X Food type interaction: Est = − 0.05, t_50.8_ = − 2.02, *p* = 0.048; significant effect of Group for high-fat foods: Est = − 0.15, t_51_ = − 2.84, *p* = 0.006, but not for low-fat foods: Est = − 0.04, t_51_ = − 1.15, *p* = 0.26).

Tastiness ratings did not change with treatment (Fig. 2c, Supplemental Table S4). Individuals with AN rated high-fat foods as less tasty than did HC (Food type X Group: Est = − 0.14, t = − 3.23, *p* = 0.002; Group: Est = − 0.13, t = − 2.51, *p* = 0.015; significant effect of Group for high-fat foods: Est = − 0.28, t_51_ = − 3.22, p = 0.002, but not for low-fat foods: Est = 0.007, t_51_ = 0.14, *p* = 0.89), and this pattern did not change over time (all other effects ps > 0.27).

#### Associations between ratings and choices

Across both timepoints Healthiness influenced Choice more among individuals with AN relative to HC (Est = 0.49, z = 3.94, *p* = 8.2 × 10^− 5^) and Tastiness influenced Choice more among HC relative to individuals with AN (Est = − 0.46, z = − 3.69, *p* = 0.0002). There was a Time by Tastiness interaction (Est = 0.17, z = 2.08, *p* = 0.037), with Tastiness influencing Choice more at Time 2 in both groups (Fig. 2d, Supplemental Table S5). Healthiness was more strongly associated with Tastiness among AN relative to HC (Est = 0.13, z = 2.19, p = 0.03; Fig. 2e, Supplemental Table S6) and the relationship between Healthiness and Tastiness ratings did not change over time (ps > 0.25).

### Neuroimaging

#### Anterior caudate

**Fig. 3:**
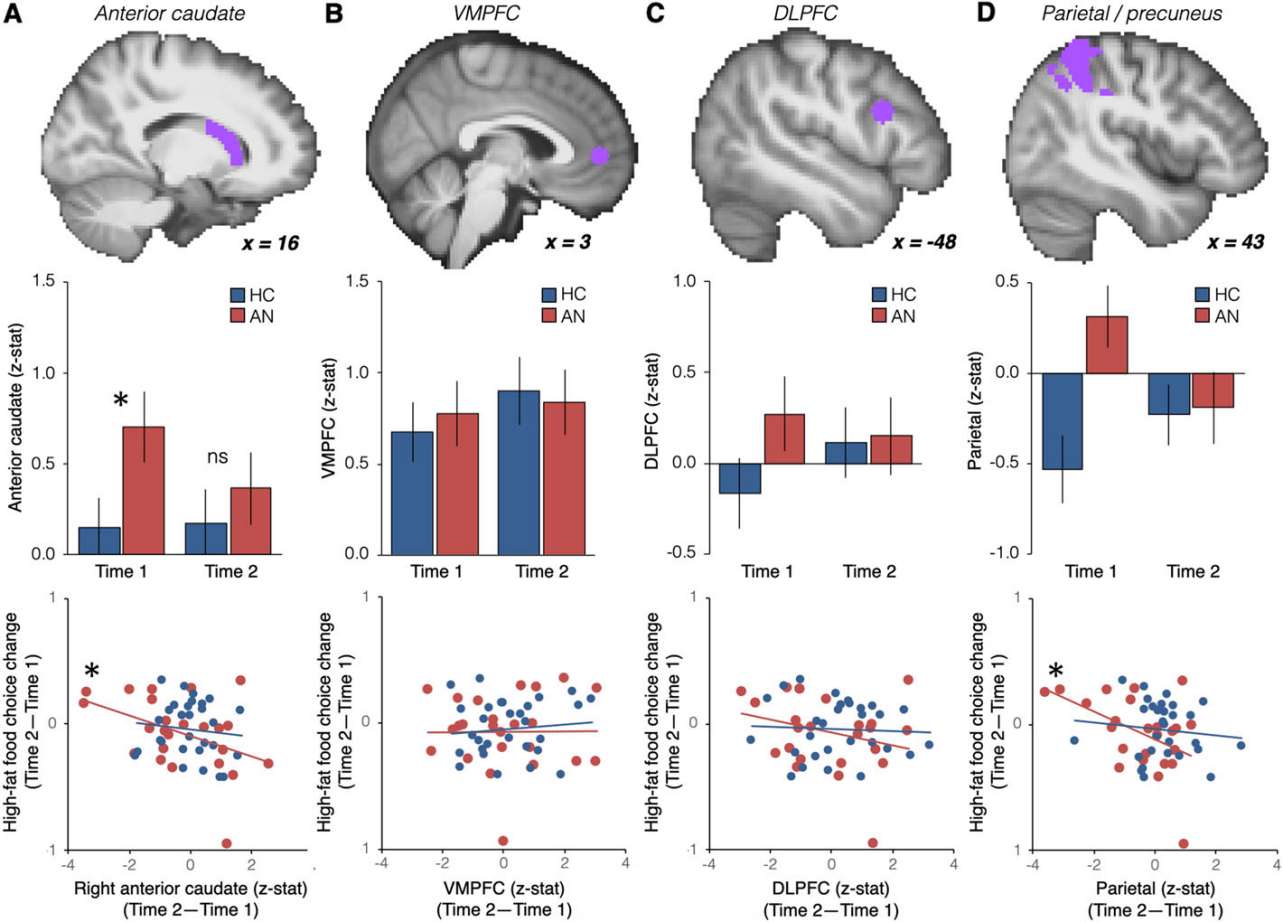
Choice-related engagement in regions of interest before and after treatment and relationship with eating behavior. **a** Values extracted from the parametric choice analysis in our a priori anatomical ROI in the right anterior caudate (top panel). Choice-related activation in the caudate differed significantly between HC and AN at Time 1, but not at Time 2 (middle panel). Change in activity in the caudate from Time 1 to Time 2 was significantly correlated with the change in proportion high-fat food choices on the food choice task among individuals with AN, but not HC. Robust regression was used due to the presence of outliers. **b** Same analyses as in **a** for the a priori VMPFC ROI (MNI = [3 51 3]; top panel). **c** Same analyses as in **a** for the a priori DLPFC ROI (MNI = [− 48 15 24]; top panel). **d** For illustration purposes the same analyses as in **a** are presented for the parietal region identified in exploratory whole-brain analyses

During the Choice phase there was no significant interaction between Group and Time in a rmANOVA (F [1, 51]=1.19, *p* = 0.28) nor a main effect of Time (F [1, 51]=0.87, *p* = 0.36). The main effect of Group approached statistical significance (F [1, 51]=2.997, *p* = 0.089) and groups differed significantly during Choice at Time 1 (t_51_ = − 2.05, *p* = 0.045). In contrast, group differences at Time 2 were not significant (t_51_ = − 0.694, *p* = 0.49; Fig. 3a. During Healthiness and Tastiness ratings there were no effects of Group or Time (ps > 0.05; Supplemental Figure S1A).

We next examined whether change in caudate activation over time was related to changes in choice behavior in AN. Among individuals with AN, increased choice of high-fat foods was associated with decreased activation of the caudate during the Choice block (r_22_ = − 0.427, *p* = 0.037, robust regression: *p* = 0.036; Fig. 3a). This relationship between neural activation and behavior was not found among the HC (r_27_ = − 0.11, *p* = 0.57; Fig. 3a). Changes in choices of low-fat foods were not related to caudate engagement in either group (AN: r_22_ = − 0.08, *p* = 0.70; HC: r_27_ = − 0.02, *p* = 0.90).

#### Prefrontal cortex activity and food choice

As in previous studies [12, 19], there was significant choice-related activation in the VMPFC ROI. That activation did not differ between groups (F [1, 51]=0.007, *p* = 0.93) or across time (F [1, 51]=0.57, *p* = 0.45) nor was there a significant interaction (F [1, 51]=0.19, *p* = 0.67; Fig. 3b). We additionally explored whether the DLPFC, previously implicated in the use of self-control for food choices [12], was differentially engaged across groups and time, but found no significant differences (Time: F [1, 51]=0.16, *p* = 0.69; Group: F [1, 51]=1.32, *p* = 0.26; Group x Time: F [1, 51]=1.00, *p* = 0.32; Fig. 3c). There were no effects of Group or Time during Healthiness and Tastiness ratings (ps > 0.05; Supplemental Figure S1BC).

Whole-brain analyses to explore areas outside a priori ROIs revealed a group difference at Time 1 in the superior parietal lobule/precuneus, with greater choice related engagement in the AN than the HC group (Supplemental Figure 2). To examine changes over time in the parietal region, we extracted values at Time 2 from the cluster identified at Time 1. There were no group differences at Time 2 (t_51_ = − 0.15, *p* = 0.88; Group X Time Interaction: F [1, 51]=6.67, *p* = 0.013; Fig. 3d). For completeness, as for the anterior caudate, we explored the relationship between changes from Time 1 to Time 2 in the parietal region and high-fat food choice changes and found a significant correlation (r_22_ = − 0.476, *p* = 0.019; robust regression: *p* = 0.022; Fig. 3d). Changes over time in the anterior caudate and parietal lobe were significantly correlated in the AN group (r = 0.745, *p* < 0.001) but not in the HC group (r = 0.338, *p* = 0.073); the difference in correlation between groups was significant (*p* = 0.038).

Whole-brain analyses of the Choice phase (Supplemental Figure S2), Healthiness rating phase (Supplemental Figure S3), and Tastiness rating phase (Supplemental Figure S4) did not reveal any other regions that changed between Time 1 and Time 2 or differed between groups.

## Discussion

This study tested whether neural mechanisms associated with maladaptive food choice in AN change with acute weight restoration. The fMRI findings during a Food Choice Task immediately following hospital admission were consistent with prior results, with greater choice-related engagement of the anterior caudate among individuals with AN. Increased selection of high-fat foods following weight restoration was associated with decreased choice-related caudate activation. On average, however, patients with AN did not substantially increase their selection of high-fat foods from Time 1 to Time 2, consistent with a prior study demonstrating the persistence of dietary restriction after treatment [9]. Additionally, whole brain analyses identified choice-related activation in a parietal/precuneus region that normalized with weight restoration. Change over time in this region was also associated with food-choice change and with caudate change. These findings underscore the association between restrictive eating and choice-related activation of the caudate in AN and suggest this brain-behavior link may be a target for treatment development.

The lack of change in choice of high-fat foods for a snack after ~ 2.5 months of hospital care is disappointing, though not surprising. The challenge of changing eating behavior, even during inpatient treatment, has been demonstrated in laboratory meal studies [8, 9] and is consistent with the high rate of relapse following hospital discharge. The ability to select a diet higher in energy density at the end of hospital care is associated with better treatment outcomes 1 year later [4, 50], suggesting that interventions that effect change in dietary patterns are likely necessary to decrease relapse. The caudate region implicated here is important for action control and choice, in general [51]. Change in maladaptive behavior in AN may depend on therapeutic techniques that focus directly on the difficult problem of changing food choices. A habit reversal based approach that targeted cues for maladaptive eating routines was successful in changing behavior in AN in a pilot study [52]. Similarly, exposure therapies that emphasize finer-grained focus on maladaptive eating have also shown promise [53]. Neuromodulation of the dorsal frontostriatal system using transcranial magnetic stimulation (TMS) showed some impact in decreasing excessive self-control over food choices among individuals with chronic AN [20]. Targeting the brain systems identified here may amplify the effects of existing treatments for AN.

In addition to the a priori ROI analyses that largely confirmed hypotheses about the caudate in food-based decision making in AN, exploratory whole-brain analyses suggested that the brain-behavior link also includes parietal/precuneus regions. The parietal lobe has been implicated in decision making and choice [54, 55]. In addition, parietal cortex is often considered part of a cognitive control network (fronto-cingulo-parietal system) [56–58], with functions that could be relevant for food choice (e.g., selective attention, response inhibition, etc.). The precuneus has been shown to play a role in a variety of higher cognitive functions, including self-referential processing and episodic memory retrieval [59]. Abnormalities in precuneus activation among individuals with AN have been identified several studies that include food related tasks [60–62] and, notably, is the most highly implicated region associated with the term ‘anorexia nervosa’ on the Neuroquery meta-analysis tool [63]. While the present study design does not allow for inferences about the specific contribution to decision-making, the findings suggest that further investigation of these cognitive processes is warranted. Although, reverse inference of cognitive processes based on brain activity is of limited utility, one hypothesis potentially worth investigating based on differential engagement of the precuneus among AN and HC, is that individuals with AN rely on memory retrieval differently than do HC; the role of episodic memories in decision making among individuals with AN has not been carefully examined. These cortical regions might also be targets for neuromodulation.

There are limited data about brain systems that contribute to the persistence of AN, especially as few studies have examined neural systems before and after treatment. The mean age and duration of illness in this study is representative of a general sample of treatment seeking adults, and is within the range seen in across neuroimaging research [64]. However, duration of illness has not been systematically considered in neuroimaging studies in AN, and may influence brain findings [65]. Existing longitudinal data, across different imaging modalities, have indicated that renourishment is associated with some brain changes. For example, structural brain abnormalities, such as gray matter volume decreases and cortical thinning, are seen among individuals with AN at low weight and tend to normalize with weight restoration [66–68]. However, studies of white matter abnormalities have not consistently reported normalization with weight restoration [69, 70]. One task-based fMRI study indicated that learning related response abnormalities in the striatum normalized after weight restoration [71]. Two studies of monetary decision-making found increases in neural activity in the striatum [72] and the dorsal anterior cingulate after weight restoration [73]. These studies together indicate that the brain is altered by starvation (and by renourishment) and that fronto-striatal circuits are among regions that undergo change across multiple studies and modalities. The stability of the neural response in the HC group in the present study supports the utility of the longitudinal assessments and strengthens the ability to draw inferences from the changes seen in the AN group. And, unlike prior longitudinal research, the present study directly examined treatment-associated changes in neural mechanisms of restrictive eating - the behavioral disturbance most directly connected to relapse, morbidity, and mortality. These findings highlight the value of explicitly linking brain and behavior by using a task demonstrated to capture maladaptive eating behavior in AN.

## Limitations

The current results should be viewed in light of several limitations. First, the sample size was modest, and it is possible that there was insufficient power to detect the full effect of treatment on neural circuits. Small sample size is a common limitation in studies of AN and suggests a need for standardization of methods such that studies can be pooled. Second, patients were studied at the end of acute inpatient treatment, shortly after full weight restoration. While weight restoration is a necessary first step in treatment of AN, it is not associated with full resolution of psychological and behavioral symptoms and may not be the optimal moment to assess brain changes in AN. On the other hand, it has been suggested that neural changes should precede behavioral effects, hopefully permitting understanding of the neural mechanisms of treatment response [74]. Third, examination of brain regions that may become engaged only after weight restoration was limited by a priori regional hypotheses. It may be that the ROIs selected (VMPFC and DLPFC) were not the most relevant or sensitive to change and, in this small study, we were unable to detect other regions of increased activation in a whole brain analysis.

## Conclusions

By linking brain and behavior, this study confirmed previous findings that during active decisions about what to eat, acutely ill individuals with AN differed from HC in engagement of the anterior caudate. The change (decrease) in caudate activation after treatment was associated with an increased tendency to choose high-fat foods, and similar activation patterns occurred in the parietal/precuneus region. Additional research is needed to determine whether food choice behavior—and associated neural mechanisms—change with longer-term remission.

## Supplementary Information

**Additional file 1.**

## Data Availability

The datasets analyzed during the current study are available from the corresponding author on reasonable request. The food choice task stimuli and nutritional content are publicly available at Columbia Academic Commons (10.7916/d8-497c-2724) and Open Science Framework (https://osf.io/483mx/); information included in Lloyd et al., 2020 [17].

## Abbreviations

AN: Anorexia nervosa
BMI: Body mass index
BOLD: Blood-oxygen level dependent
DLPFC: Dorsolateral prefrontal cortex
GLM: General linear model
HC: Healthy control volunteer
ITI: Intertrial interval
NYSPI: New York State Psychiatric Institute
VMPFC: Ventromedial prefrontal cortex
IQ: Intelligence quotient
DICOM: Digital Imaging and Communications in Medicine
NIFTI: Neuroimaging Informatics Technology Initiative
FSL: FMRIB Software Library
MNI: Montreal Neurological Institute
FD: Framewise Displacement
