# Supplementary material for: Changes in brain and behavior during food-based decision-making following treatment of anorexia nervosa
Source: J Eat Disord. 2021 Apr 17;9:48. doi: 10.1186/s40337-021-00402-y (PMC8052661; doi:10.1186/s40337-021-00402-y)
Supplement: Supplementary file 1 — Additional file 1. [file 40337_2021_402_MOESM1_ESM.docx]

**SUPPLEMENTAL MATERIALS FOR**

Brain and Behavior Changes in Food-Based Decision-Making with Treatment of Anorexia Nervosa

**Contents of supplement:**

**Table S1** Number of dropped volumes from fMRI analyses

**Other tables?**

**Table S2.** Analysis of Choice (Accompanies Figure 2A)

**Table S3.** Analysis of Healthiness ratings (Accompanies Figure 2B)

**Table S4.** Analysis of Tastiness ratings (Accompanies Figure 2C)

**Table S5.** Analysis of Healthiness and Tastiness rating influence on Choice (Accompanies Figure 2D)

**Table S6.** Analysis of Healthiness rating influence on Tastiness ratings (Accompanies Figure 2E)

**Figure S1.** Healthiness and Tastiness rating-related activity in ROI.

**Figure S2**. Whole-brain analyses of Choice phase.

**Figure S3**. Whole-brain analyses of Healthiness rating phase.

**Figure S4**. Whole-brain analyses of Tastiness rating phase.

**Table S1.** Number of dropped volumes from fMRI analyses

|  | **HC** | | | **AN** | | |  |  |
| --- | --- | --- | --- | --- | --- | --- | --- | --- |
|  | ***M*** | ***SD*** | ***Median*** | ***M*** | ***SD*** | ***Median*** | ***t*** | ***p*** |
| **Time 1** |  |  |  |  |  |  |  |  |
| Run 1 | 1.41 | 4.60 | 0 | 3.38 | 7.60 | 0 | -1.16 | 0.25 |
| Run 2 | 2.55 | 7.60 | 0 | 3.42 | 5.34 | 0 | 0.27 | 0.79 |
| Run 3 | 3.10 | 7.48 | 0 | 2.57 | 6.69 | 0 | -0.62 | 0.54 |
| **Time 2** |  |  |  |  |  |  |  |  |
| Run 1 | 2.25 | 5.04 | 0 | 6.39 | 15.25 | 0 | -0.47 | 0.641 |
| Run 2 | 4.38 | 9.61 | 0 | 6.00 | 9.30 | 0 | -1.35* | 0.22 |
| Run 3 | 3.45 | 9.61 | 0 | 10.54 | 15.33 | 2 | -1.97* | 0.06 |

* Welch's t-test due to non-equality of variances.

*Food Choice Task behavior analyses*

Rating and choice data, and relationships between ratings and choices, were analyzed using multilevel regression models, including Group (HC/AN), Food type (low-fat/high-fat), and Time (Time 1/Time 2) as independent variables (see Methods).

**Table S2.** Analysis of Choice (Accompanies Figure 2A): Group (HC/AN) X Food type (Low-fat/High-fat X Time (Time 1/Time 2)

|  | ***Estimate*** | ***SE*** | ***z-value*** | ***P*** |
| --- | --- | --- | --- | --- |
| Intercept | 0.26 | 0.18 | 1.42 | 0.15 |
| Food type | -0.37 | 0.14 | -2.60 | 0.009 |
| Time | -0.15 | 0.11 | -1.40 | 0.16 |
| Group | -0.42 | 0.15 | -2.87 | 0.004 |
| Food type X Time | 0.00 | 0.04 | 0.07 | 0.95 |
| Food type X Group | -0.42 | 0.09 | -4.72 | 2.36E-06 |
| Time X Group | -0.12 | 0.11 | -1.10 | 0.27 |
| Food type X Time X Group | 0.02 | 0.04 | 0.52 | 0.60 |

**Table S3.** Analysis of Healthiness ratings (Accompanies Figure 2B): Group (HC/AN) X Food type (Low-fat/High-fat X Time (Time 1/Time 2)

|  | ***Estimate*** | ***SE*** | ***t-value*** | ***P*** |
| --- | --- | --- | --- | --- |
| Intercept | 2.99 | 0.12 | 25.79 | < 2.00E-16 |
| Food type | -0.75 | 0.11 | -6.70 | 2.60E-09 |
| Time | 0.01 | 0.02 | 0.41 | 0.68 |
| Group | -0.10 | 0.04 | -2.41 | 0.0195 |
| Food type X Time | 0.01 | 0.02 | 0.96 | 0.34 |
| Food type X Group | -0.05 | 0.02 | -2.02 | 0.048 |
| Time X Group | 0.03 | 0.02 | 1.84 | 0.07 |
| Food type X Time X Group | 0.03 | 0.02 | 1.62 | 0.11 |

**Table S4.** Analysis of Tastiness ratings (Accompanies Figure 2C): Group (HC/AN) X Food type (Low-fat/High-fat X Time (Time 1/Time 2)

|  | ***Estimate*** | ***SE*** | ***t-value*** | ***P*** |
| --- | --- | --- | --- | --- |
| Intercept | 3.37 | 0.08 | 44.18 | <2.00E-16 |
| Food type | -0.04 | 0.07 | -0.60 | 0.5 |
| Time | 0.01 | 0.03 | 0.44 | 0.66 |
| Group | -0.13 | 0.05 | -2.51 | 0.015 |
| Food type X Time | 0.00 | 0.01 | 0.06 | 0.9 |
| Food type X Group | -0.14 | 0.04 | -3.23 | 0.002 |
| Time X Group | 0.03 | 0.03 | 1.11 | 0.27 |
| Food type X Time X Group | -0.01 | 0.01 | -0.79 | 0.43 |

**Table S5.** Analysis of Healthiness and Tastiness rating influence on Choice across Group (HC/AN) and Time (Time 1/Time 2) (Accompanies Figure 2D)

|  | ***Estimate*** | ***SE*** | ***z-value*** | ***P*** |
| --- | --- | --- | --- | --- |
| (Intercept) | 0.31 | 0.24 | 1.31 | 0.19 |
| Time | -0.23 | 0.17 | -1.37 | 0.17 |
| Group | -0.66 | 0.22 | -2.96 | 0.003 |
| Health | 0.85 | 0.13 | 6.44 | 1.23E-10 |
| Taste | 1.93 | 0.13 | 15.09 | < 2.00E-16 |
| Health X Time | 0.11 | 0.06 | 1.82 | 0.069 |
| Health X Group | 0.49 | 0.12 | 3.94 | 8.17E-05 |
| Time X Group | -0.16 | 0.17 | -0.98 | 0.33 |
| Taste X Time | 0.17 | 0.08 | 2.08 | 0.037 |
| Taste X Group | -0.46 | 0.13 | -3.69 | 0.0002 |
| Health X Time X Group | -0.01 | 0.06 | -0.17 | 0.87 |
| Taste X Time X Group | 0.08 | 0.08 | 1.03 | 0.30 |

**Table S6.** Analysis of Healthiness rating influence on Tastiness ratings across Group (HC/AN) and Time (Time 1/Time 2) (Accompanies Figure 2E)

|  | ***Estimate*** | ***SE*** | ***t-value*** | ***P*** |
| --- | --- | --- | --- | --- |
| (Intercept) | 3.37 | 0.08 | 44.35 | < 2.00E-16 |
| Time | 0.01 | 0.03 | 0.37 | 0.71 |
| Group | -0.14 | 0.05 | -2.55 | 0.014 |
| Health | 0.48 | 0.06 | 7.88 | 7.55E-11 |
| Health X Time | 0.02 | 0.02 | 0.96 | 0.34 |
| Health X Group | 0.13 | 0.06 | 2.19 | 0.03 |
| Time X Group | 0.03 | 0.03 | 1.09 | 0.28 |
| Health X Time X Group | 0.01 | 0.02 | 0.54 | 0.59 |

*Imaging of Healthiness and Tastiness*


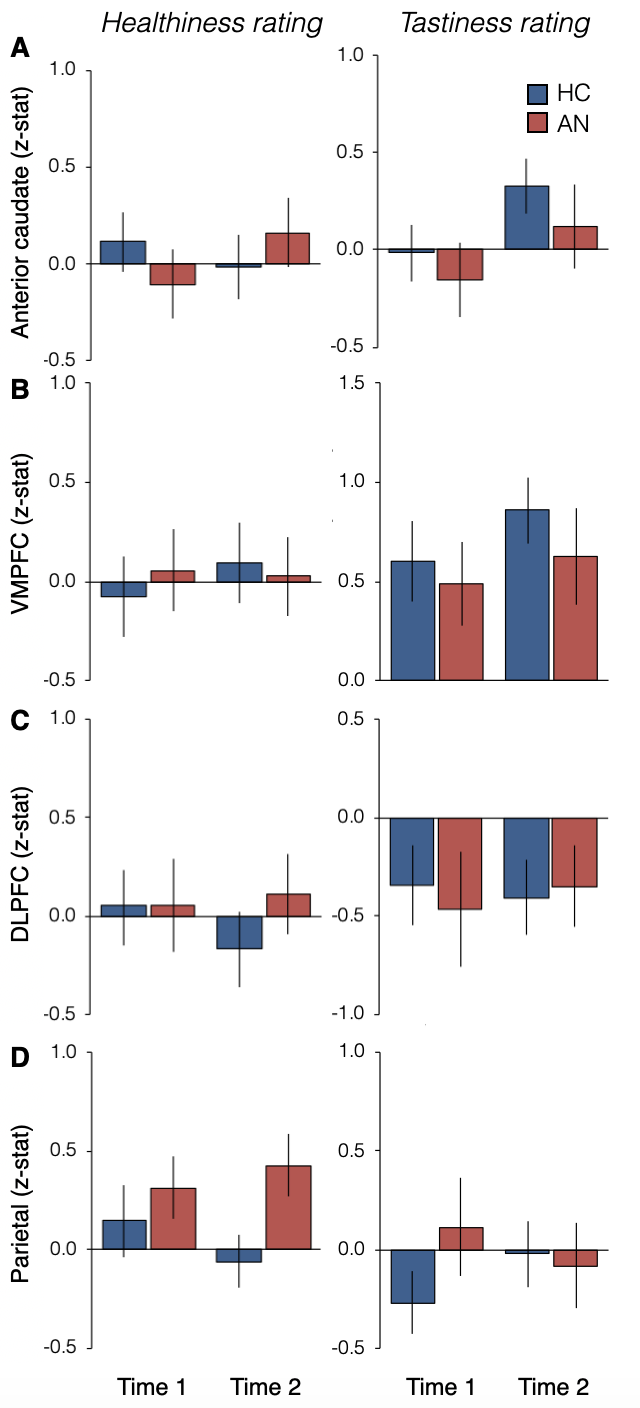


**Figure S1.** Rating-related activity in ROIs. Values extracted from the parametric analysis during Healthiness (left column) and Tastiness (right column) ratings. There were no group differences or changes over time in these regions.

(**A**) anterior caudate. (**B**) ventromedial prefrontal cortex (vmPFC). (**C**) dorsolateral prefrontal cortex (dlPFC). (**D**) Parietal region from whole-brain Choice phase analysis.


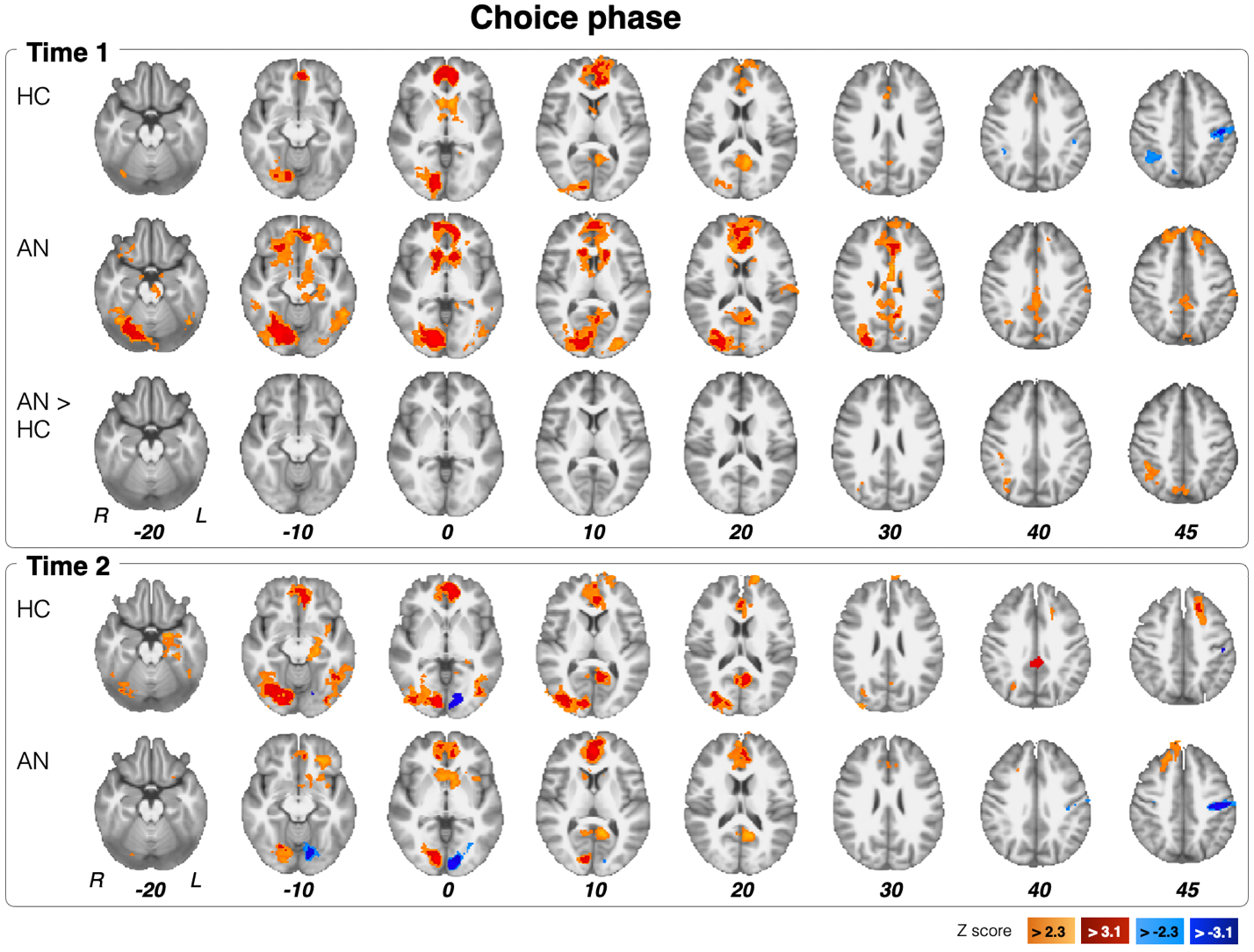
**Figure S2.** Whole-brain analyses of the Choice phase. Regions correlated with choice values in a parametric analysis shown separately for HC and AN groups at Time 1 and Time 2. Group and Time comparisons only shown for significant contrasts. Maps were family-wise error (FWE)-corrected p<0.05, whole-brain, cluster-forming threshold Z>3.1 (darker colors) and Z>2.3 (lighter colors).


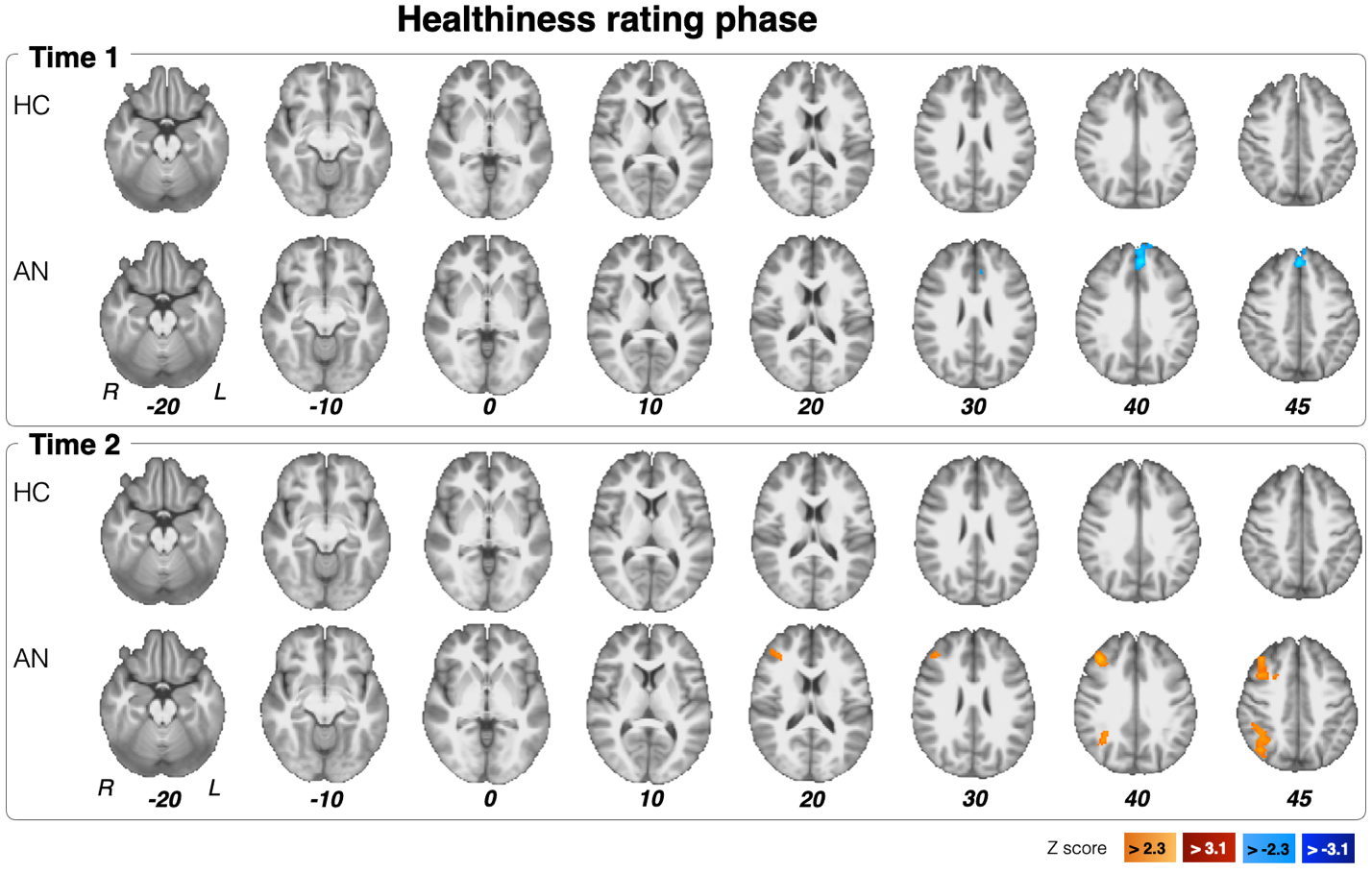


**Figure S3.** Whole-brain analyses of the Healthiness rating phase. Regions correlated with healthiness ratings in a parametric analysis shown separately for HC and AN groups at Time 1 and Time 2. Maps were family-wise error (FWE)-corrected p<0.05, whole-brain, cluster-forming threshold Z>3.1 (darker colors) and Z>2.3 (lighter colors).


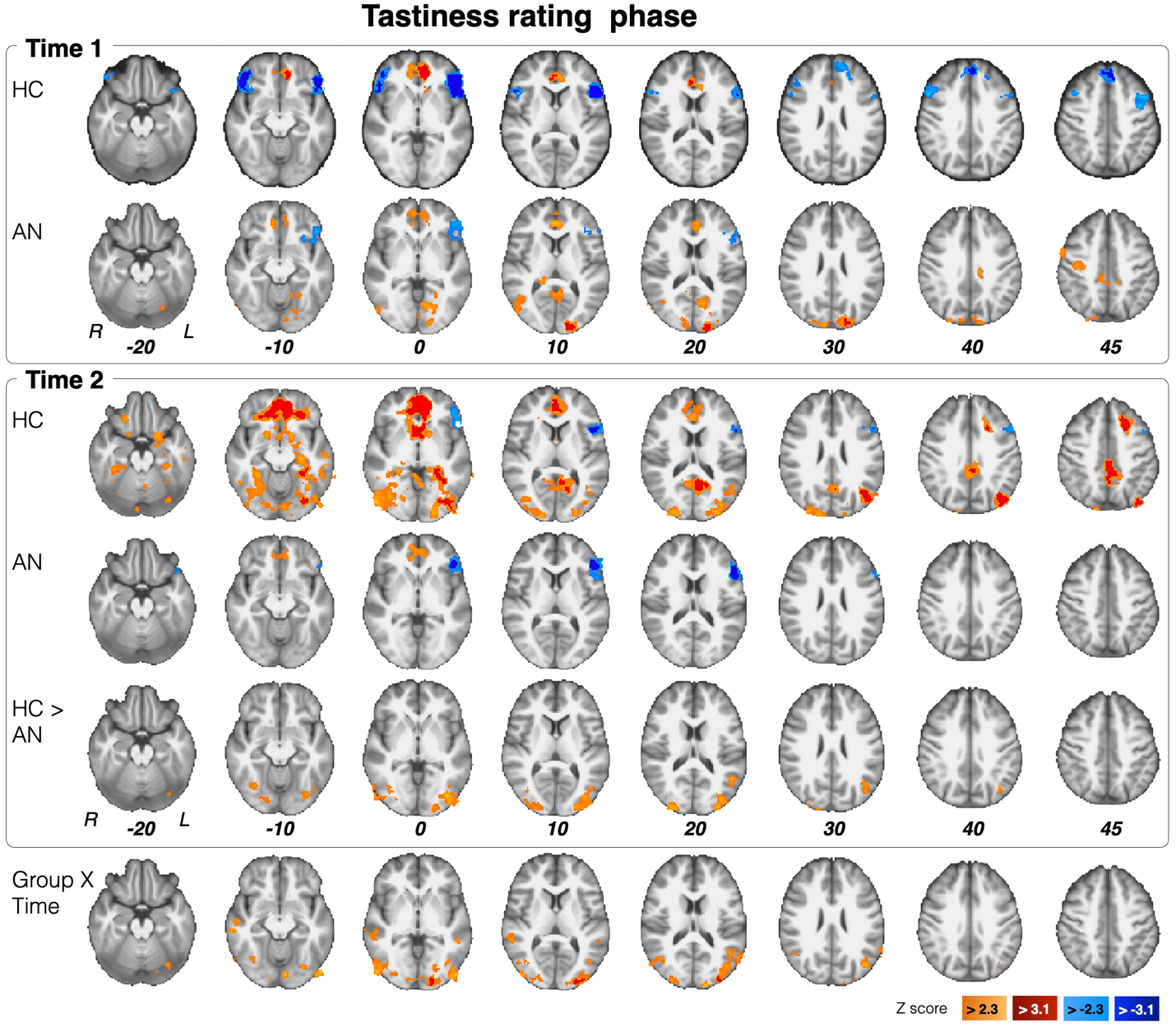


**Figure S4.** Whole-brain analyses of the Tastiness rating phase. Regions correlated with tastiness ratings in a parametric analysis shown separately for HC and AN groups at Time 1 and Time 2. Group and Time comparisons only shown for significant contrasts. Maps were family-wise error (FWE)-corrected p<0.05, whole-brain, cluster-forming threshold Z>3.1 (darker colors) and Z>2.3 (lighter colors).
